# Effect of Curcumin in Experimental Pulmonary Tuberculosis: Antimycobacterial Activity in the Lungs and Anti-Inflammatory Effect in the Brain

**DOI:** 10.3390/ijms23041964

**Published:** 2022-02-10

**Authors:** Jacqueline V. Lara-Espinosa, María Fernanda Arce-Aceves, Manuel O. López-Torres, Vasti Lozano-Ordaz, Dulce Mata-Espinosa, Jorge Barrios-Payán, Carlos Alfredo Silva-Islas, Perla D. Maldonado, Brenda Marquina-Castillo, Rogelio Hernández-Pando

**Affiliations:** 1 Sección de Patología Experimental, Instituto Nacional de Ciencias Médicas y Nutrición Salvador Zubirán, Mexico City 14080, Mexico; mariferarce@ciencias.unam.mx (M.F.A.-A.); lopeztorresmanuel88@gmail.com (M.O.L.-T.); loov1288@hotmail.com (V.L.-O.); dulmat@yahoo.com.mx (D.M.-E.); qcjbp77@yahoo.com.mx (J.B.-P.); brenda.marquinac@incmnsz.mx (B.M.-C.); 2 Laboratorio de Patología Vascular Cerebral, Instituto Nacional de Neurología y Neurocirugía Manuel Velasco Suárez, Mexico City 14269, Mexico; charlssilv@gmail.com (C.A.S.-I.); maldonado.perla@quimica.unam.mx (P.D.M.)

**Keywords:** tuberculosis, curcumin, neuroinflammation, antibacterial

## Abstract

Tuberculosis (TB) is one of the ten leading causes of death worldwide. Patients with TB have been observed to suffer from depression and anxiety linked to social variables. Previous experiments found that the substantial pulmonary inflammation associated with TB causes neuroinflammation, neuronal death, and behavioral impairments in the absence of brain infection. Curcumin (CUR) is a natural product with antioxidant, anti-inflammatory and antibacterial activities. In this work, we evaluated the CUR effect on the growth control of mycobacteria in the lungs and the anti-inflammatory effect in the brain using a model of progressive pulmonary TB in BALB/c mice infected with drug-sensitive mycobacteria (strain H37Rv). The results have shown that CUR decreased lung bacilli load and pneumonia of infected animals. Finally, CUR significantly decreased neuroinflammation (expression of TNFα, IFNγ and IL12) and slightly increased the levels of nuclear factor erythroid 2-related to factor 2 (Nrf2) and the brain-derived neurotrophic factor (BDNF) levels, improving behavioral status. These results suggest that CUR has a bactericidal effect and can control pulmonary mycobacterial infection and reduce neuroinflammation. It seems that CUR has a promising potential as adjuvant therapy in TB treatment.

## 1. Introduction

Tuberculosis (TB), the oldest human pandemic, generally caused by infection via the lung with *Mycobacterium tuberculosis* (*Mtb*), remains the foremost cause of death among bacterial infectious diseases [1,2]. Bacillus Calmette–Guérin (BCG), a live attenuated strain of *Mycobacterium bovis* [3], the primary cause of bovine TB [4], is the only approved vaccine against TB and is the most widely used vaccine in history [4,5]. Unfortunately, though billions of individuals were vaccinated in the past century, TB remains a severe threat to global health [5]. In 2019, 10 million persons developed TB, and approximately 1.4 million people died of this infection (208,000 were HIV-infected). Due to the COVID-19 pandemic, the number of deaths attributable to TB is expected to increase to similar levels presented in 2012, increasing to between 200,000 and 400,000 deaths (1.6–1.8 million deaths) [6]. The COVID-19 pandemic and related lockdown restrictions significantly impacted providing and monitoring TB surveillance strategies globally [7].

The typical treatment for pulmonary TB comprises two months of fourfold therapy with isoniazid (INH), rifampicin (RMP), ethambutol (EMB), and pyrazinamide (PZA) followed immediately by an additional four months of dual administration of RMP and INH [8]. This regimen is well known and has been generally adopted worldwide for decades, and about 85% of patients will have a successful treatment outcome [9]. However, this treatment frequently produces side effects (gastric, neurologic and haematological alterations). Furthermore, the long duration affects patient adherence, resulting in treatment abandonment, which results in the emergence of drug-resistant TB [10,11,12]. In addition, there has been a rapidly emerging problem of multidrug-resistant (MDR) TB, which is frequently lethal, extremely expensive and complicated to treat [13]. A fluoroquinolone (moxifloxacin, levofloxacin) can be employed in patients with monoresistance to RMP or INH. The duration of treatment is then raised to a total of 6 to 9 months (INH resistance) or 18 to 20 months (RMP resistance), depending on the individual course [14].

Although new antibiotics against TB have been developed in recent years, there is still a need to discover new antituberculous agents that are effective in treating MDR TB cases and novel agents that can shorten the long conventional chemotherapy drug-sensitive TB. Within this context, new synthetic drugs and natural products from medicinal plants are potential sources of new antimycobacterial products [15].

TB is a severe chronic systemic lung disease, although *Mtb* can spread to other organs, causing extrapulmonary disease [16]. Inflammation is a response to infection, antigen challenge, or tissue injury designed to eradicate microbes or injury agents and potentiate tissue repair [17]. However, excessive inflammation leads to tissue damage and can, if severe, cause physiological decompensation, organ dysfunction and death [17,18]. Perturbations in host tissue homeostasis triggered by infectious microorganisms, such as *Mtb*, activate immune surveillance mechanisms that promote inflammation. Infection with *Mtb* consists of several phases, which begins with phagocytosis of bacteria by macrophages and progress towards a TH1 lymphocyte response that fights the bacteria and causes tissue damage by excessive inflammation [19,20]. Systemic inflammation occurs in pulmonary and extrapulmonary TB diseases and is characterized by increased concentrations of inflammatory molecules such as acute-phase proteins, lipid mediators (e.g., prostaglandin E2 [PGE2]), several pro-inflammatory cytokines, and chemokines [21].

The central nervous system (CNS) was long considered as a site of restricted immune surveillance due to the absence of lymphatic vessels, the blood–brain barrier (BBB), and slow transplant rejection [18]. The CNS is a compartmentalized organ, including the parenchyma, the ventricles comprising the choroid plexus and cerebrospinal fluid (CSF), the meningeal layers that enclose the parenchyma, and various “absolute” (BBB) and semi-permeable barriers (blood CSF, blood-leptomeningeal) [22]. The level of steady-state immune privilege varies considerably among these compartments [22]. During neuroinflammation, the immune landscape of the CNS changes dramatically; resident immune cells become activated, and the inflammatory leukocytes can infiltrate the parenchyma [22]. This process could be helpful by protecting the brain from pathogens and neurotoxic agents and supporting tissue repair processes [23]. However, neuroinflammation is an essential feature of many neurodegenerative diseases such as multiple sclerosis (MS), Alzheimer’s disease (AD), Parkinson’s disease (PD), narcolepsy, as well as psychiatric diseases and behavioral disorders such as schizophrenia, autism, and depression [24,25].

Another problem related to neuroinflammation is psychiatric disorders, including depression and anxiety [26]. Lung diseases are among chronic medical conditions strongly associated with psychiatric disorders [27,28]. Evidence shows that anxiety, depression and emotional distress participate in the incapacity generated by TB, and they are related to the severity of symptoms, the number of reported symptoms, higher rates of health services use, short treatment compliance, more comprehensive course of treatment, reduced control of the disease and death [29]. Epidemiological evidence establishes a relationship between depression, anxiety and TB [30]. For instance, in an Afghan study, 69.55% of the MDR-TB patients with HIV-negative status presented with significant levels of depression [31]. A South African study determined that 81.1% of the TB patients presented with depression and 31.9% with anxiety [32]. A recent study in India determined that 80.37% of TB patients had depression and 74% anxiety [33]. A Brazilian study found a 60.2% increase in depression in individuals with pulmonary TB [34]. Patients with a more prolonged disease have a higher incidence of depression and anxiety [35], and depression is higher in patients with pulmonary than in extrapulmonary TB [33]. An important aspect is that TB patients still suffer from depression even under treatment, anxiety scores remain high [36,37], and adult patients are more susceptible to depression [38]. Additionally, MDR-TB patients present higher levels of depression [31,39,40,41]. Furthermore, TB patients show low-to-moderate rates of suicidal ideation (9.0%) and a record of suicide attempts (3.1%) [42].

Even though the link between TB, depression and anxiety has not been clearly understood [30], it seems that pro-inflammatory cytokines that are highly produced by the tuberculous lungs can reach the brain by specific carrier-mediated transport mechanisms. Furthermore, these cytokines are also overproduced in the brain during this peripheral inflammatory process by binding to their receptors in the endothelial cells and nerve cells in the circumventricular organs and other brain areas lacking the BBB [43]. In addition, pro-inflammatory cytokines such as interferon-gamma (IFNγ), IFNα and tumor necrosis factor (TNFα) contribute to the development of depressive disorder by regulating neuronal excitability, reducing the levels of serotonin and causing changes in other mechanisms of neurotransmission and neuronal signaling in brain regions involved with depression, oxidative injury, and hippocampal neuronal damage [44,45,46]. Therefore, in TB patients, *Mtb* infection’s peripheral inflammation generated in the lung could induce CNS inflammation and neuropsychiatric disorders, such as depression and anxiety.

In diseases with an inflammatory component, first-line medications have traditionally been agents that reduce inflammation [47]. Curcumin (CUR) or diferuloylmethane (1, 7-bis (4-hydroxy-3-methoxyphenol)-1, 6-heptadiene-3, 5-dione) is a polyphenolic compound obtained from the rhizomes of *Curcuma longa* [48,49], a rhizomatous native plant from South and Southeast Asia that belongs to the family Zingiberaceae [50]. Research has revealed that CUR has pleiotropic properties, including anti-inflammatory, antioxidant, chemopreventive, chemotherapeutic activity, neuroprotective properties, and antibacterial activity [50,51,52,53]. The pleiotropic actions of CUR are derived from its complex chemistry and its ability to influence multiple signaling pathways [50]. CUR controls the inflammatory response by downregulating the activity of the enzymes cyclooxygenase-2 (COX-2), lipoxygenase, and inducible nitric oxide synthase (iNOS). In addition, CUR suppresses the activation of nuclear factor kappa B (NF-κB) activation; inhibits the production of the inflammatory cytokines TNF-α, interleukin (IL)-1, -2, -6, -8, and -12, monocyte chemoattractant protein (MCP) and migration inhibitory protein; and down-regulates mitogen-activated and Janus kinases [54]. In addition, CUR protects the brain from damage through the upregulated expression of the transcription factor, the nuclear factor erythroid 2-related to factor 2 (Nrf2) expression [55], and the hippocampal levels of brain-derived neurotrophic factor (BDNF) [56].

We have demonstrated neuroinflammation and distinct neuropsychiatric abnormalities in an experimental model of progressive pulmonary TB without brain infection [57]. Therefore, we hypothesize that CUR administration could decrease the pulmonary bacilli burdens and neuroinflammation with its behavioral abnormalities in TB mice. Thus, the present study aimed to evaluate the efficacy of the administration of CUR on lung disease evolution, neuroinflammation, the Nrf2 and BDNF expression and behavioral alterations in a murine model of pulmonary TB.

## 2. Results

### 2.1. The Effect of Curcumin (CUR) Treatment on Survival, Bacilli Loads and Tissue Damage (Pneumonia) in Experimental Pulmonary Tuberculosis

**Figure 1:**
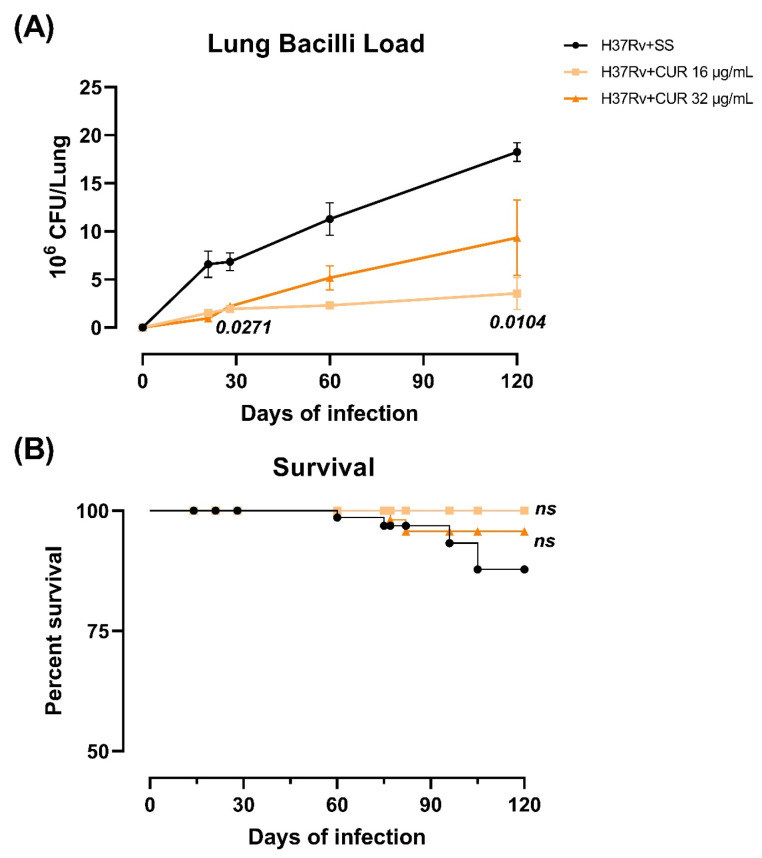
The effect of curcumin (CUR) therapy (16 or 32 μg/mL) given early in the TB infection (14 days post-infection) on the progression of lung disease in intratracheal infected mice with 2.5 × 10^5^ *Mtb* colony-forming units (CFU) virulent strain H37Rv. (**A**) Bacillary burdens in the lungs of TB mice treated with CUR and control mice who only received saline solution (SS) as the vehicle. F (1.221, 2.442) = 21.66, *p* = 0.0287, two-way analysis of variance (ANOVA). Tukey’s multiple comparisons test. (**B**) Survival rates of control mice given SS versus CUR-treated animals (*n* = 36). *p* = 0.2464, logrank test for trend. Data are presented as mean ± SEM. Both doses of CUR significantly decreased the bacterial load in the lung from day 21 post-infection and tended to increase the survival rate. However, the 16 μg/mL dose was more effective in decreasing lung disease than the 32 μg/mL dose. CUR administration in both doses is safe to administer in animals infected with *Mtb*, as they do not aggravate lung disease and have a beneficial effect.

**Figure 2:**
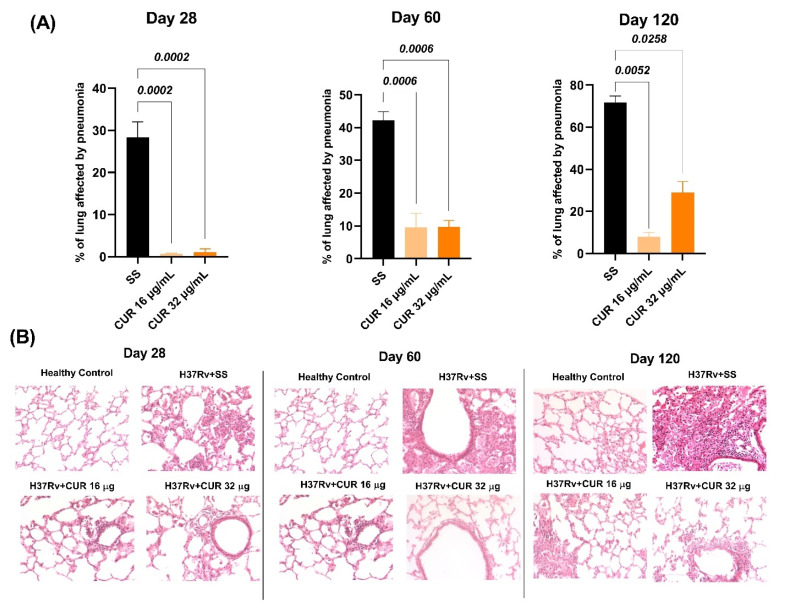
Effect of curcumin (CUR) treatment (16 μg/mL or 32 μg/mL) starting in the early TB infection (14 days post-infection) on pneumonia of infected mice with 2.5 × 10^5^ *Mtb* colony-forming units (CFU) virulent strain H37Rv. (**A**) Percentage of the pneumonic area determined by automated morphometry at 28, 60 and 120-days post-infection of control mice that only received saline solution (SS) as the vehicle, and infected animals treated with CUR. Day 28 F (2, 6) = 52.30, *p* = 0.0002, ordinary ANOVA. Dunnett’s multiple comparisons test. Day 60 F (2, 6) = 36.98, *p* = 0.0004, ordinary ANOVA. Dunnett’s multiple comparisons test. Day 120 F (1.377, 2.753) = 65.86, *p* = 0.005, ordinary ANOVA. Dunnett’s multiple comparisons test. Data are presented as mean ± SEM (*n* = 6/day/group) (**B**) Representative micrographs at 28-, 60- and 120-days post-infection of infected animals that received CUR, control mice that only received SS and healthy mice without infection. All micrographs at 20× magnification, hematoxylin/eosin staining. The results show extensive pneumonic areas in the control group, while there are fewer pneumonic areas in the other groups, particularly in mice receiving CUR 16 μg/mL.

Tuberculous animals were given CUR (16 or 32 μg/mL) via an intraperitoneal route starting on day 14 after infection to see how these treatments affected the progression of lung disease in BALB/c mice after endotracheal infection with a high dose of the *Mtb* H37Rv strain, trying to find a dose that reduced neuroinflammation without aggravating lung disease. We evaluated the effect of CUR during the infection, determining bacillary loads in the lungs and brains, the *Mtb*-infected animals’ survival and pneumonia. Infected mice treated with CUR showed that both doses significantly decreased the lungs’ bacillary load after day 21 post-infection compared to control animals. However, the 16 μg/mL dose was more effective in reducing the lung bacilli load (Figure 1A). None of the treatments caused the growth of mycobacteria in the brain. In addition, the survival rate of animals that received CUR slightly improved compared to the control non-treated group (Figure 1B). These findings correlated with the morphometric analysis; there was a significant decrease in the lung area affected by pneumonia in mice treated with CUR than the control group (Figure 2).

These results suggest that CUR does not aggravate lung disease; on the contrary, it has a beneficial effect, making it safe to administer in animals infected with *Mtb.* Furthermore, we observed that the 16 μg/mL dose was more effective than the 32 μg/mL dose in decreasing lung disease. Hence, in the remainder of the experiments detailed in this article, we only used the 16 μg/mL dose.

### 2.2. The Effect of CUR Treatment on Cytokine Expression in Distinct Brain Areas of TB Mice

**Figure 3:**
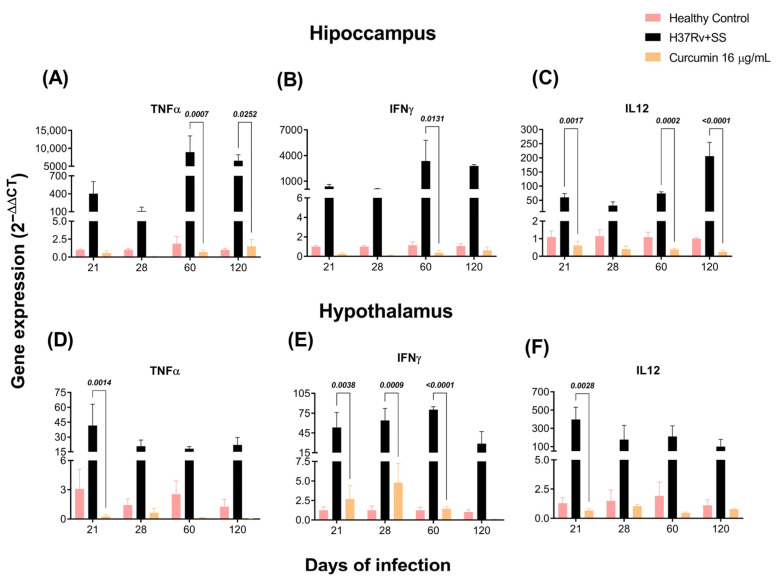
Effect of curcumin (CUR) treatment (16 μg/mL) on pro-inflammatory cytokines of the hippocampus and hypothalamus of mice infected with 2.5 × 10^5^ *Mtb* virulent strain H37Rv colony-forming units (CFU), mice that received saline solution (SS) as vehicle and mice that were not infected and were used as controls. (**A**) TNFα expression on the hippocampus. F (2, 6) = 7.327, *p* = 0.0245. Mixed-effects model (REML). Tukey’s multiple comparisons test. (**B**) IFNγ expression on the hippocampus. F (2, 6) = 4.459, *p* = 0.0651. REML. Tukey’s multiple comparisons test. (**C**) IL-12 expression on the hippocampus. F (3, 8) = 76.07, *p* < 0.0001. REML. Tukey’s multiple comparisons test. (**D**) TNFα expression on the hypothalamus. F (2, 21) = 14.05, *p* = 0.0001. REML. Tukey’s multiple comparisons test. (**E**) IFNγ expression on the hypothalamus. F (2, 6) = 29.59, *p* = 0.0008. REML. Tukey’s multiple comparisons test (**F**) IL-12 expression on the hypothalamus. F (2, 21) = 10.64, *p* = 0.0006. REML. Tukey’s multiple comparisons test. RNA was extracted from hippocampal and hypothalamic homogenates, reverse-transcribed to cDNA, and gene expression changes of the appropriate cytokine were examined. The fold-change data were adjusted to the healthy controls’ expression levels. Data are presented as mean ± SEM (*n* = 6/day/group). There was a significant increase in gene expression in the absence of detectable brain infection from day 21 post-infection. The treatment with CUR decreased the expression of these pro-inflammatory cytokines in the hippocampus mainly at days 60 and 120 post-infection. In the hypothalamus, the treatment with CUR decreased TNFα and IFNγ at days 60 and 120 post-infection, although there was a tendency of reduction after day 21. The expression of IL12 decreased after day 21.

**Figure 4:**
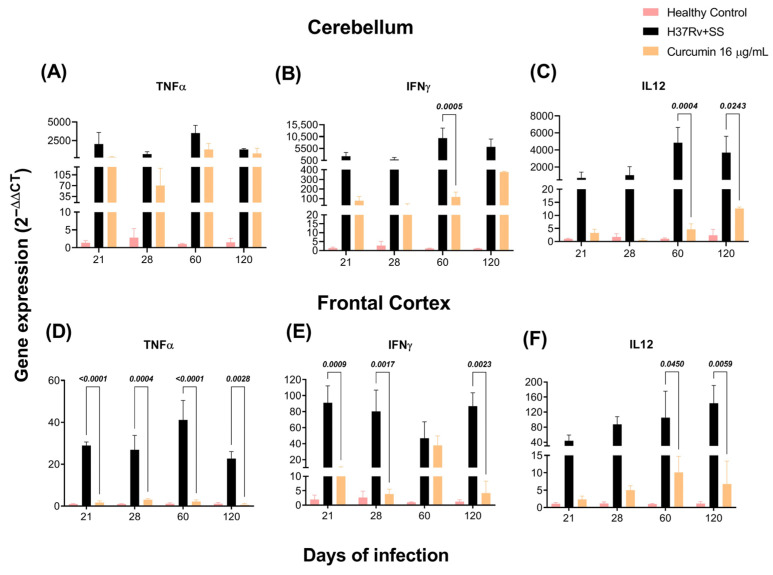
Effect of curcumin (CUR) treatment (16 μg/mL) on pro-inflammatory cytokines of the cerebellum and frontal cortex of mice infected with 2.5 × 10^5^ *Mtb* colony-forming units (CFU) virulent strain H37Rv, mice that received saline solution (SS) as the vehicle, and mice that were not infected and were used as controls. (**A**) TNFα expression on the cerebellum. F (2, 6) = 4.211, *p* = 0.0720. REML (**B**) IFNγ expression on the cerebellum. F (2, 6) = 7.322, *p* = 0.0245. REML. Tukey’s multiple comparisons test (**C**) IL-12 expression on the cerebellum. F (2, 21) = 14.39, *p* = 0.0001. REML. Tukey’s multiple comparisons test. (**D**) TNFα expression on the frontal cortex. F (2, 22) = 76.20, *p* < 0.0001. REML. Tukey’s multiple comparisons test (**E**) IFNγ expression on the frontal cortex. F (2, 22) = 33.71, *p* <0.0001. REML. Tukey’s multiple comparisons test (**F**) IL-12 expression on the frontal cortex. F (2, 6) = 6.175, *p* = 0.0350. REML. Tukey’s multiple comparisons test. RNA was extracted from cerebellar homogenates, reverse-transcribed to cDNA, and gene expression changes of the appropriate cytokine were examined. The fold-change data were adjusted to the healthy controls’ expression levels. Data are presented as mean ± SEM (*n* = 6/day/group). There was a significant increase in gene expression in the absence of detectable brain infection after day 21 post-infection. The treatment with CUR decreased the expression of IFNγ and IL12 in the cerebellum at days 60 and 120 post-infection. In the frontal cortex, the treatment with CUR decreased TNFα and IFNγ after day 21 post-infection and IL12 at days 60 and 120 post-infection.

As previously observed, the brains of mice with TB produce pro-inflammatory cytokines without bacterial growth [57]. By RT-PCR, we investigated the effects of CUR vs. saline solution on TNFα, IFNγ, and IL-12 expression in the hippocampus, hypothalamus, cerebellum, and frontal cortex of mice infected with *Mtb*. Our results showed that the animals treated with CUR considerably decreased the expression of these cytokines in the hippocampus at days 60 and 120 post-infection (Figure 3A–C). In the hypothalamus, the results showed that CUR induced a trend to decrease the expression of pro-inflammatory cytokines from day 21 post-infection (Figure 3D–F). In the cerebellum, expression of IFNγ and IL12 decreased at days 60 and 120 post-infection, and TNFα remained high (Figure 4A–C). Finally, in the frontal cortex, CUR significantly reduced the expression of these pro-inflammatory cytokines from day 21 post-infection. Interestingly, the most important effect of CUR decreasing the inflammatory process was observed in the frontal cortex (Figure 4D–F) starting at day 21 post-infection. In contrast, in the hippocampus, hypothalamus and cerebellum, the effect was primarily observed in the advanced phase of the lung infection.

### 2.3. The Effect of CUR Treatment after Early TB Infection on Diverse Behavioral Abnormalities

**Figure 5:**
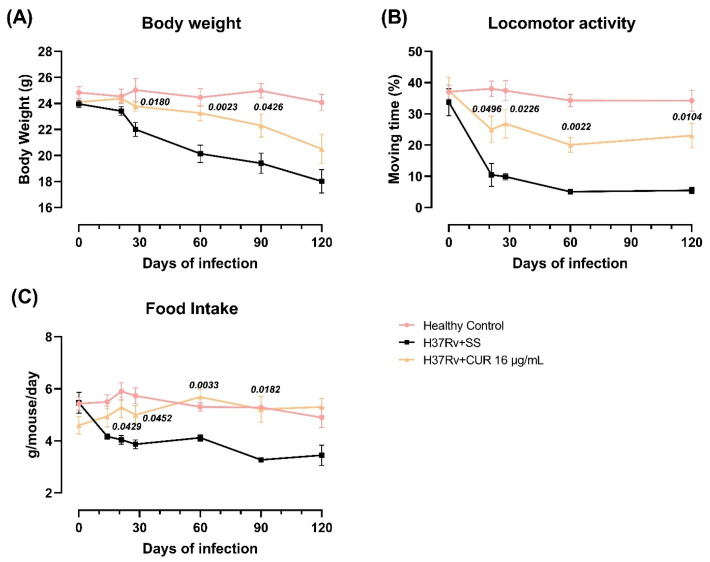
Effect of curcumin (CUR) treatment (16 μg/mL) on sickness behavior in TB mice. (**A**) Bodyweight loss of infected animals that received CUR, control mice that only received saline solution (SS) as the vehicle, and healthy mice without infection. F (2, 400) = 42.71, *p* < 0.0001. Two-way ANOVA. Dunnett’s multiple comparisons test. (**B**) Locomotor activity of infected animals that received CUR, control mice that only received SS as the vehicle, and healthy mice without infection. F (2, 15) = 69.23, *p* < 0.0001. Two-way ANOVA. Dunnett’s multiple comparisons test. (**C**) Food intake of infected animals that received CUR, control mice that only received SS as the vehicle, and healthy mice without infection. F (2, 15) = 13.72, *p* < 0.0004. Two-way ANOVA. Dunnett’s multiple comparisons test. Data are presented as mean ± SEM (*n* = 6/day/group). The treatment with CUR decreased the sickness behavior of infected animals. There was an improvement in body weight, increased locomotor activity, and increased food intake.

Previous experiments showed that pulmonary infection with *Mtb* induced sickness behavior, expressed by a significant reduction in body weight, locomotor activity and food intake in infected animals [57]. Sickness behavior is a response associated with the inflammatory process. We determined the effect of CUR administration during early *Mtb* infection on sickness behavior [58]. The results showed that the treatment with CUR decreased TB mice’s sickness behavior (Figure 5). There was a slight increase in body weight, mostly at 28, 60 and 90-days post-infection (Figure 5A). Locomotor activity (LMA) and food intake were also considerably improved after one week of treatment (Figure 5B,C).

**Figure 6:**
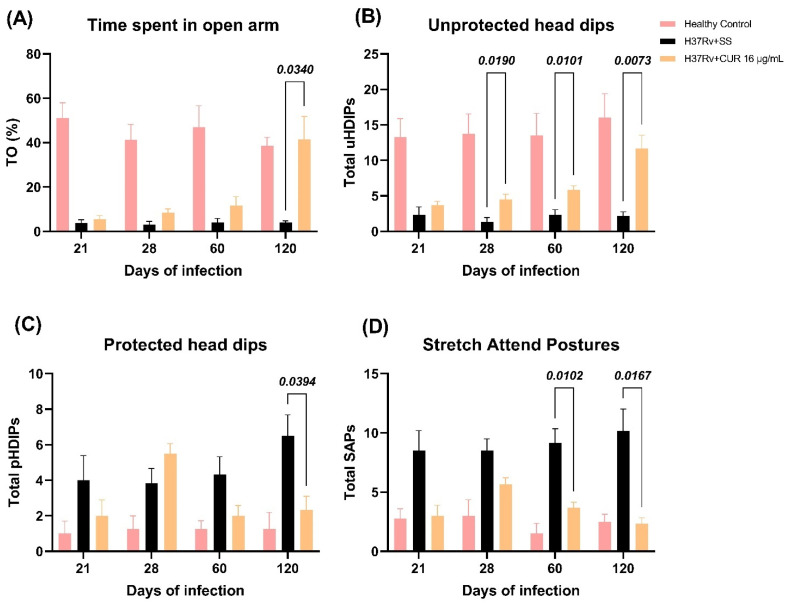
Effect of curcumin (CUR) treatment (16 μg/mL) on anxiety-like behavior in TB mice evaluated in the elevated I-maze. (**A**) Time spent on the open arm by infected mice who received CUR, control mice that just received the saline solution (SS) as the vehicle, and healthy mice who were not infected (%TO). F (2, 13) = 74.40, *p* < 0.0001. Tukey’s multiple comparisons test. (**B**) Unprotected head dips (uHdips) of infected mice given CUR, control mice given only SS as the vehicle, and healthy mice not infected. F (2, 13) = 65.32, *p* < 0.0001. Two-way ANOVA. Tukey’s multiple comparisons test. (**C**) Protected head dips (pHDIPS) of infected mice who received CUR, control mice that just received SS as the vehicle, and healthy mice with no infection. F (2, 13) = 10.24, *p* = 0.0021. Two-way ANOVA. Tukey’s multiple comparisons test. (**D**) Stretched attend postures (SAP) of infected animals received CUR, control mice that only received SS as the vehicle, and healthy mice without infection. F (2, 13) = 39.81, *p* < 0.0001. Two-way ANOVA. Tukey’s multiple comparisons test. Data are presented as mean ± SEM (*n* = 6/day/group). CUR produced a significant increase in %TO at day 120 post-infection, an increase in uHdips from day 28 post-infection, a decrease in pHDIPS, and a decrease in SAP compared to the saline-treated mice at days 60 and 120 post-infection. All these data suggest that CUR has an anxiolytic-like activity on TB mice.

Anxiety is another essential behavioral change observed in animals infected with *Mtb* and TB patients. We found that lung inflammation produced anxiogenic behavior in our model of pulmonary TB [57]. Therefore, we evaluated the CUR treatment’s effect on infected animals that showed anxiety-like behavior using the elevated I-maze [59]. We observed that the treatment with CUR increased the time spent by mice on the open arm (TO) at day 120 post-infection (Figure 6A), increased the unprotected head dips (uHDIPS) (scanning over the sides of the maze downward towards the floor from uncovered open arm by the animal) from day 28 post-infection (Figure 6B), reduced the protected head dips (pHDIPS) (scanning by the animal over the sides of the maze downward towards the floor from protected area) at day 120 post-infection (Figure 6C), and stretched attend postures (SAP) (forward elongation of the body when the animal was either standing still or moving slowly forward) at days 60 and 120 post-infection (Figure 6D). Thus, the treatment with CUR showed an anxiolytic-like activity on TB mice, mainly in the late phase of infection.

**Figure 7:**
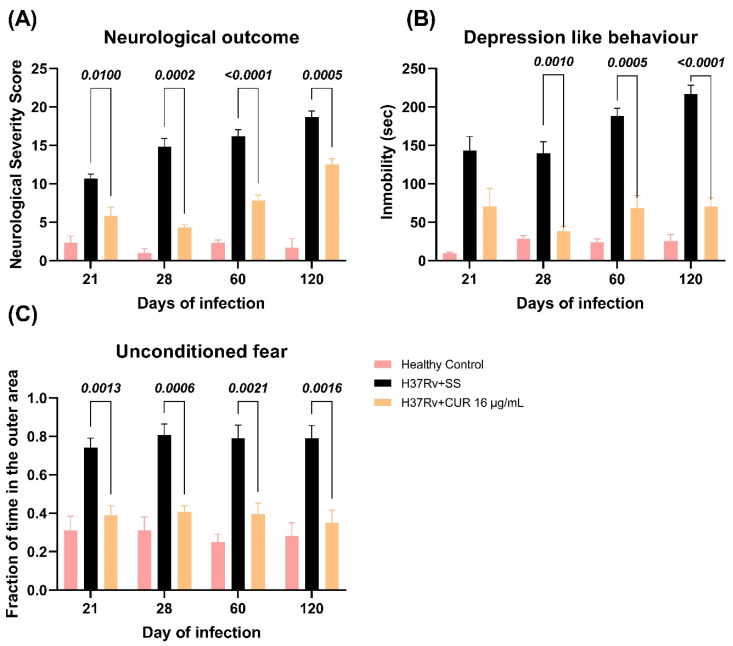
Effect of curcumin (CUR) treatment (16 μg/mL) on neurological damage, depression-like behavior and unconditioned fear of TB mice. (**A**) Neurological outcome of mice treated with CUR, control mice that only received saline solution (SS) as the vehicle, and healthy mice without infection. F (2, 12) = 142.2, *p* < 0.0001. Two-way ANOVA. *p* < 0.0001. Dunnett’s multiple comparisons test. (**B**) Depression-like behavior of mice treated with CUR, control mice that only received SS as the vehicle, and healthy mice without infection. F (2, 12) = 154.6, *p* < 0.0001. Two-way ANOVA. Dunnett’s multiple comparisons test. (**C**) Unconditioned fear of mice treated with CUR, control mice that only received SS as the vehicle, and healthy mice without infection. F (2, 15) = 45.82, *p* < 0.0001. Two-way ANOVA. Dunnett’s multiple comparisons test. Data are presented as mean ± SEM (*n* = 6/day/group). The treatment with CUR decreased neurological damage from day 21 post-infection and decreased the depression-like behavior of infected animals from day 28. The treatment decreased the unconditioned fear from day 21.

**Figure 8:**
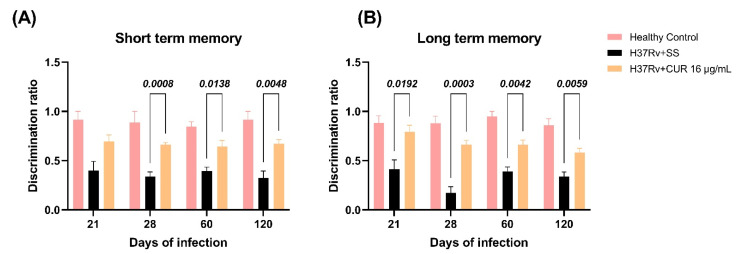
Effect of curcumin (CUR) treatment (16 μg/mL) on TB mice’s memory damage. (**A**) Short-term memory of mice treated with CUR, control mice that only received saline solution (SS) as the vehicle, and healthy mice without infection. F (2, 15) = 77.03, *p* < 0.0001. Two-way ANOVA. Dunnett’s multiple comparisons test. (**B**) Long-term memory of mice treated with CUR, control mice that only received SS as the vehicle, and healthy mice without infection. F (2, 12) = 86.60, *p* < 0.0001. Two-way ANOVA. Dunnett’s multiple comparisons test. Data are presented as mean ± SEM (*n* = 6/day/group). Animals with TB treated with CUR showed a significant improvement in short-term memory from day 28 post-infection and long-term memory from day 21.

We have previously shown that pulmonary infection with *Mtb* induces depression-like behavior, neurological impairment, and unconditioned fear [57]. In the present study, we evaluated the treatment with CUR on these behavioral changes. The results showed that CUR decreased the neurological damage from day 21 post-infection (Figure 7A). Similar results were seen in the depression-like behavior, which was considerably reduced from day 28 post-infection by decreased immobility time compared to vehicle-treated controls (Figure 7B). Furthermore, the unconditioned fear decreased from day 21 post-infection (Figure 7C). These results suggest CUR has an anti-depressive effect and reduces unconditioned fear, and the neurological status of the sick animals was improved due to the treatment. Previous experiments showed that pulmonary infection with *Mtb* induces damage in short-term memory from day 14 post-infection and long-term memory from day 1 post-infection [36]. The treatment with CUR enhanced the short-term memory from day 28 post-infection (Figure 8A) and long-term memory from day 21 post-infection (Figure 8B) of TB mice in the object recognition test. These results indicate a higher memory performance in CUR-treated TB mice.

### 2.4. The Effect of CUR Treatment on Nrf2 and BDNF Levels in the Frontal Cortex and Hippocampus of TB MICE

**Figure 9:**
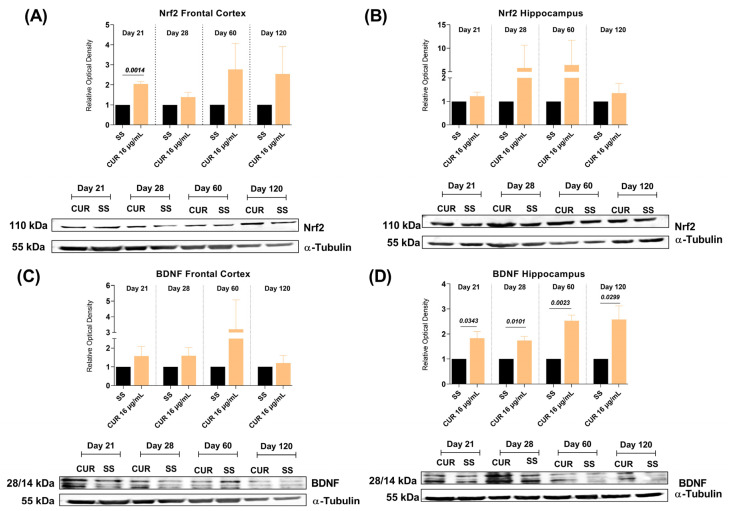
Effect of curcumin (CUR) treatment (16 μg/mL) on the Nrf2 and BDNF levels in the frontal cortex and hippocampus of mice infected with *Mtb* H37Rv and control mice that just received the saline solution (SS) as the vehicle. (**A**) Quantification of the relative optical density of Nrf2 in the frontal cortex. Day 21, t = 11.55, df = 3. Unpaired *t*-test. The lower panel shows a representative image of the western blot at 21, 28, 60, and 120-days post-infection. (**B**) Quantification of the relative optical density of Nrf2 in the hippocampus. Unpaired *t*-test. The lower panel shows a representative image of the western blot at 21, 28, 60 and 120-days post-infection. (**C**) Quantification of the relative optical density of BDNF in the frontal cortex. Unpaired *t*-test. The lower panel shows a representative image of the western blot at 21, 28, 60 and 120-days post-infection. (**D**) Quantification of the relative optical density of BDNF in the hippocampus. Day 21, t = 3.157, df = 4. Unpaired *t*-test. Day 28, t = 4.587, df = 4. Unpaired *t*-test. Day 60, t = 6.945, df = 4. Unpaired *t*-test. Day 120, t = 6.166, df = 6. Unpaired *t*-test. The lower panel shows a representative image of the western blot at 21, 28, 60 and 120-days post-infection. Data are presented as mean ± SEM (*n* = 6/day/group). The treatment with CUR increased Nrf2 levels in the frontal cortex, importantly at day 21 post-infection, showed an increasing trend at day 60 and 120 post-infection, and slightly increased Nrf2 levels in the hippocampus from day 21 post-infection. The treatment with CUR slightly increased BDNF levels in the frontal cortex from day 21 post-infection and, importantly, increased BDNF levels in the hippocampus at days 21, 28, 60 and 120 post-infection.

Oxidative stress and neuroinflammation are linked with cognitive decline and neuronal damage [60]. It is reported that various antioxidant enzymes are regulated by Nrf2, which protects cells from oxidative damage and inflammation [61]. Hence, we evaluated the effects of CUR on the Nrf2 expression in the frontal cortex and hippocampus of TB mice, two areas where we observed a significant decrease in pro-inflammatory cytokines and which are related to cognitive and behavioral processes. The results demonstrated that the treatment with CUR increased Nrf2 levels in the frontal cortex, importantly at day 21 post-infection, and there was an increasing trend at day 60 and 120 post-infection (Figure 9A). In addition, the treatment with CUR slightly increased Nrf2 levels in the hippocampus from day 21 post-infection (Figure 9B). These results suggest that treatment with CUR protects the cells of the cortex and hippocampus from oxidative damage and inflammation, which could be related to the improvement in the behavioral state of animals with TB.

Since BDNF has been implicated in the anti-depression effects and improvement in memory produced by several drugs [62,63], we evaluated the effects of CUR on BDNF in the frontal cortex and hippocampus of TB mice. Our results showed that CUR treatment slightly increased BDNF levels in the frontal cortex from day 21 post-infection (Figure 9C). Furthermore, the therapy with CUR significantly increased BDNF levels in the hippocampus at days 21, 28, 60 and 120 post-infection (Figure 9D). These results could be linked to the beneficial effect CUR presented on the behavioral changes of animals with TB, which improved memory and decreased depression-like behavior.

These results suggest that the administration of CUR reduces the bacilli load in the lung, pneumonia, neuroinflammation, behavioral abnormalities, and slightly increases Nrf2 and BDNF in the murine model of experimental pulmonary TB.

## 3. Discussion

Previous work showed neuroinflammation and neuropsychiatric abnormalities in an experimental model of progressive pulmonary TB without brain infection [57]. In this work, we evaluated the effect of CUR in lung disease evolution and the capability of CUR to inhibit both the pro-inflammatory events induced by *Mtb* on the CNS and the behavioral abnormalities.

TB is the leading cause of death amongst all bacterial diseases, accounting for more than 1.4 million fatalities per year [64]. The emergence of multidrug-resistant (MDR) *Mtb* strains is an increasing problem requiring novel treatment options [65]. Our results showed that the treatment with 16 or 32 μg/mL of CUR reduced the bacilli lung load of mice infected with the drug-sensitive *Mtb* H37Rv. The decrease in bacilli lung load was related to reduced pneumonia and improved survival. These results agree with previous studies showing that CUR is a potential anti-*Mtb* agent [65]. It has been demonstrated that CUR has a minimum concentration inhibitory (MIC) activity of 16 μg/mL against drug-susceptible *Mtb* H37Rv, isoniazid-resistant *Mtb* H37Rv, rifampicin-resistant H37Rv, streptomycin-resistant H37Rv and ethambutol-resistant H37Rv [66]. The effect of CUR against *Mtb* could be direct, affecting the bacterial lipid metabolism since lipids and their metabolism is critical to all aspects of mycobacterial infection, pathogenicity, and persistence in the host [66]. CUR can also inhibit bacterial intracellular growth and promote sensible drug strain clearance, as demonstrated in differentiated THP-1 human monocytes, primary human alveolar macrophages, and Raw 264.7 cells infected with *Mtb* H37Rv or MDR clinical isolates [67,68], by promoting caspase-3-dependent apoptosis and autophagy.

In antigen-presenting cells (APCs) infected with H37Rv, CUR nanoparticles improved autophagy costimulatory activity promoting the generation of inflammatory cytokines and other mediators [69]. In H37Rv-challenged mice, nanocurcumin improves the effectiveness of the BCG vaccination by inducing long-lasting central memory T cells (TCM) of the Th1 and Th17 lineages [69]. CUR’s anti-inflammatory properties have also been used to increase the efficacy of previously authorized anti-microbial drugs through synergistic effects [70]. CUR also protected BALB/c mice against *Klebsiella pneumonie*-induced lung inflammation [71], which coincides with our results that showed a significant decrease in the pneumonia area of TB mice treated with CUR. It seems that CUR can directly damage *Mtb*, promote bacterial killing in the cytoplasm of macrophages, and has an immunomodulatory effect, which agrees with our results of efficient mycobacterial clearance in the lungs.

We have demonstrated that *Mtb* pulmonary infection caused neuroinflammation and behavioral abnormalities in the absence of bacteria in the brain [57]. Neuroinflammation can be caused by an increased peripheral inflammatory response and oxidative stress, activating microglia that contribute to brain pathology [72]. Therefore, the present work evaluated the effect of CUR in the inflammatory response in the hippocampus, hypothalamus, cerebellum and frontal cortex. The results showed a significant effect of CUR on the pro-inflammatory cytokines TNFα, IL12, and IFNγ mRNA expression levels that were considerably lower in the treated group than the control non-treated TB group. Thus, CUR has a substantial anti-inflammatory effect in the brain that constitutes a neuroprotective benefit clearly shown by our study. These results agree with various studies that have shown the anti-inflammatory effect of CUR in the CNS, administrated either as a treatment or as adjuvant therapy in several illnesses [73], such as in the prevention of a cognitive deficit induced by ethanol through modulating oxidative-nitrosative stress and decreasing the levels of pro-inflammatory cytokines (TNF, IL-1), NFκB, and caspase 3 in different brain regions of ethanol-treated rat pups [74]. CUR decreased interleukin-23 (IL-23) and interleukin-17 (IL-17) levels in the CNS in a model of retinal ischemia-reperfusion injury [75] and reduced the infarct size and the levels of IL-1, TNFα, cyclooxygenase-2 (COX-2) and PGE-2 in the cerebral ischemia model by activating the peroxisome proliferator-activated receptor gamma (PPARγ) [76]. CUR was also able to block Toll-like receptors 2 and 4 (TLR-2/4) and NF-κB in rats with persistent and localized cerebral ischemia [77]. In neurodegenerative diseases, CUR treatment reduced β-Amyloid Peptide (Aβ) deposits in the brain and improved cognitive and synaptic dysfunction in Alzheimer’s disease (AD) patients [78]. CUR also reduced the number of hypertrophic astrocytes in the hippocampus of Aβ(1-40)-treated mice, countered downregulated mRNA expression of the glial fibrillar acidic protein (GFAP) and improved the spatial memory disorders (such disorders being symptomatic in AD) [79]. Demethoxycurcumin, a CUR derivative, reduced the expression in the hippocampus of pro-inflammatory IL-1 and GFAP in a rat model of AD [80]. CUR can also impact Aβ metabolism and aggregation of the β-amyloid fibrils (fAβ) [81] and substantially inhibit IL-1, IL-6, and TNFα production in Aβ exposed microglia via the mitogen-activated protein kinase (MEK1/MEK2) p38 pathways [82]. CUR also decreased pro-inflammatory cytokines (IL-6, IL-1, and TNFα) in the 1-methyl-4-phenyl-1,2,3,6-tetrahydropyridine (MPTP) model of Parkinson’s disease (PD) and protected dopaminergic neurons from degeneration [83,84]. Another study found that CUR specifically protects axons, but not neuronal cell bodies, from NO-mediated degeneration [85]. Thus, there is considerable evidence that CUR has efficient anti-inflammatory effects that mediate neuroprotection. The present study extends the information about the therapeutical benefit of CUR by the demonstration for the first time of the prevention of neuroinflammation in experimental pulmonary TB.

Various cytokines and inflammatory factors that produce neuroinflammation also participate in the pathophysiology of neuropsychiatric disorders such as depression [86] by generating oxidative stress, affecting neurotransmitter production and even generating neuronal death [86]. Thus, we also examined the effect of CUR treatment on a range of behavioral disorders as we have demonstrated that *Mtb* infection caused behavioral abnormalities in the absence of bacteria in the brain [57]. Our results show that CUR therapy reduced sickness, reduced anxiety-like, reduced depression-like behavior, improved the neurological outcome, and enhanced short- and long-term memory in tuberculous mice. Interestingly, similar results were found in patients with obesity, where supplementation of 1 g/day of CUR had an antianxiety effect related to the antioxidant and anti-inflammatory properties of CUR [87]. Another study performed in rats subjected to immobilization stress and pre-treated with CUR (200 mg/kg/day) for seven days showed a decrease in anxiety-like behavior, depression-like behavior and improved memory function, which was related to the activity of antioxidant enzymes [88]. Similar results have been observed in Cadmium (Cd) exposure Swiss-Webster mice that received CUR (300 mg/kg). In this work, the treatment improved body weight gain and locomotor activity, decreased anxiety in the plus-maze, and increased learning capability [89]. Moreover, CUR treatment had an important suppressive effect on the cadmium-induced oxidative stress and increased the levels of serotonin (5-HT) and dopamine (DA) in the forebrain area [89].

Indeed, there is evidence that CUR has a beneficial effect on humans suffering from depression and anxiety [90,91], linked to CUR anti-inflammatory effects, dopamine release, antioxidant activity, and neurotrophic factor regulation [91]. BDNF in the hippocampus is necessary for the cognition enhancement effect of chronic CUR in an AD model [63]. Chronic CUR also resulted in a dose-dependent increase in hippocampal BDNF in a model of depression [56]. These data coincide with our results, where we observed a significant increase in BDNF levels in the hippocampus of animals with TB treated with CUR. BDNF plays a crucial role in regulating neuronal development, maintenance and survival, and cognition, formation, and storage of memories [92]. Therefore, the increase in BDNF in the hippocampus of TB animals could be related to the beneficial effect of CUR on memory and the decrease in depression-like behavior in this model. Another factor that protects the brain from injury is Nrf2, as oxidative damage plays a critical role in many central nervous system diseases [93]. CUR protected from injury in a model of an ischemic brain through the Akt/Nrf2 pathway [94]. CUR has a neuroprotective effect in a model of traumatic brain injury (TBI) associated with activating the Nrf2 pathway [95]. These data coincide with our results, as we observed a slight increase in the levels of Nrf2 in the TB animals treated with CUR. These data suggest that treatment with CUR has a beneficial effect on various neuroinflammatory and neurodegenerative diseases, including those related to pulmonary TB.

This investigation revealed the efficacy of CUR administration as a novel treatment for controlling neuroinflammation in chronic infectious diseases such as pulmonary TB. In addition, it is worth noting that CUR had a therapeutic effect on lung disease, indicating that CUR might be used as a coadjuvant treatment in TB chemotherapy.

## 4. Materials and Methods

### 4.1. Reagents and Antibodies

The Middlebrook 7H9 and 7H10 media and the OADC (oleic acid, albumin, dextrose, and catalase) were obtained from Becton-Dickinson, (Detroit, MI, USA). The RNeasy^®^ Mini Kit for RNA extraction, the Omniscript^®^ Reverse Transcription Kit for complementary DNA acquisition, and the QuantiTectTM SYBR^®^ for RT-PCR were obtained from Qiagen (Germantown, MD, USA). The primers for the cytokines studied were acquired from InvitrogenTM Thermo Fisher Scientific (Waltham, MA, USA). CUR, DMSO (Dimethylsulfoxide) and ethanol was purchased from Sigma Aldrich (Zwijndrecht, The Netherlands). Primary antibody against BDNF (ab72439) was obtained from Abcam Inc. (Cambridge, MA, USA). Primary antibody against Nrf2 (T-19; sc-30915) antibody was obtained from Santa Cruz Biotechnology (Santa Cruz, CA, USA). Secondary antibodies against rabbit (711-035-152), goat (705-035-147) and mouse (715-035-150) were purchased from Jackson ImmunoResearch Laboratories Inc. (Jennersville, PA, USA). All other reagents were analytical grade and acquired from known commercial sources.

### 4.2. Animals

Three hundred pathogen-free adult male BALB/c mice, aged eight weeks, were obtained from Mexico’s Instituto Nacional de Ciencias Médicas y Nutrición Salvador Zubirán (INCMNSZ) animal house facility. Mice were housed in groups of five (*n* = 5) and divided into two sets: healthy controls (HC, *n* = 84) and infected mice (H37Rv, *n* = 216). The distribution of animals within each experiment was made following the recommendations for the Replacement, Refinement and Reduction of Animals in Research (3R) [96] and adjusted to the total of animals provided by INCMNSZ. All efforts were made to minimize animal suffering and the number of animals used. All of the animals were housed in an approved animal holding facility with a 12:12 h light-dark cycle (lights on at 07:00 h) and a regulated temperature (23 ± 1 °C), and humidity (50–20 percent). Food and drink were freely available. All animal studies were carried out under the ARRIVE standards and Mexican Constitution statute NOM 062–Z00-1999, with consent from the INCMNSZ’s Ethical Committee for Animal Experimentation, protocol number: PAT-1865-16/19-1 (approved on 7 August 2016).

### 4.3. The Experimental Murine Model of Pulmonary TB

A mouse model of progressive pulmonary TB has previously been reported [47,97,98]. The reference *Mtb* strain H37Rv was grown in a 7H9 medium enriched with OADC. For all of the experiments, mid-log-phase cultures were used. *Mtb* were counted and stored at a temperature of −80 °C until needed. Bacterial aliquots were thawed and pulse-sonicated to eliminate clumping. After infecting mice, a portion of the bacterial inoculum was plated to validate the amount of CFU and viability of the CFU provided to the animals. Eight-week-old male BALB/c mice were anesthetized in a gas chamber with 0.1 mL sevoflurane per mouse. A blunt stainless-steel cannula was introduced through the mouth and guided to the trachea. The cannula’s appropriate intratracheal (IT) placement was confirmed by rubbing the tracheal rings with the tiny ball from the cannula. Mice were infected with 2.5 × 10^5^ live bacilli by IT installation. Mice were kept in a vertical position until they recovered spontaneously. Then, in a P-3 biosecurity level facility, a total of 184 infected mice were kept in groups of five in cages equipped with micro-isolators.

### 4.4. Experimental Design

**Figure 10:**
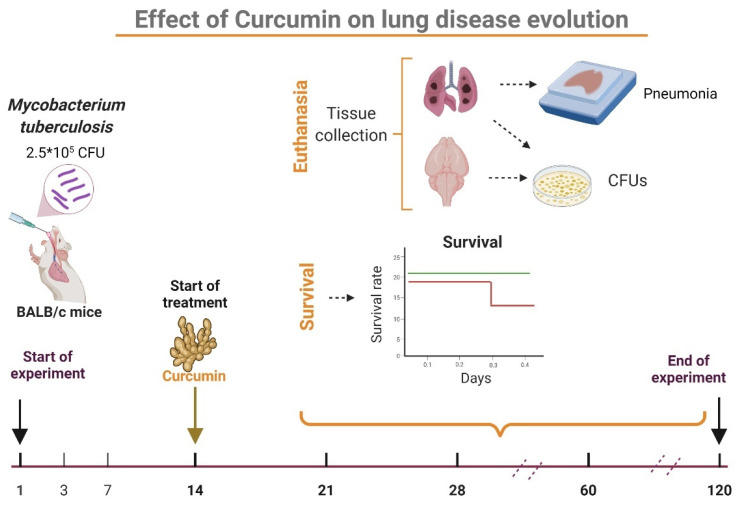
Experimental design to evaluate curcumin (CUR) therapy on the progression of lung disease. Eight-week-old BALB/c mice were infected with 2.5 × 10^5^ live and viable *Mtb* H37Rv. The therapy with CUR began on day 14. We administered two CUR doses: 16 or 32 μg/mL, and a group was treated with a saline solution (SS) as control. Post-infection animals were euthanized on days 21, 28, 60, and 120, and the brain and lungs were taken to assess the bacillary burdens and pneumonia area. In addition, we assessed the animal’s survival during the whole experiment. Each experimental age group’s samples were examined individually (created with BioRender.com, accessed on 27 June 2021).

**Figure 11:**
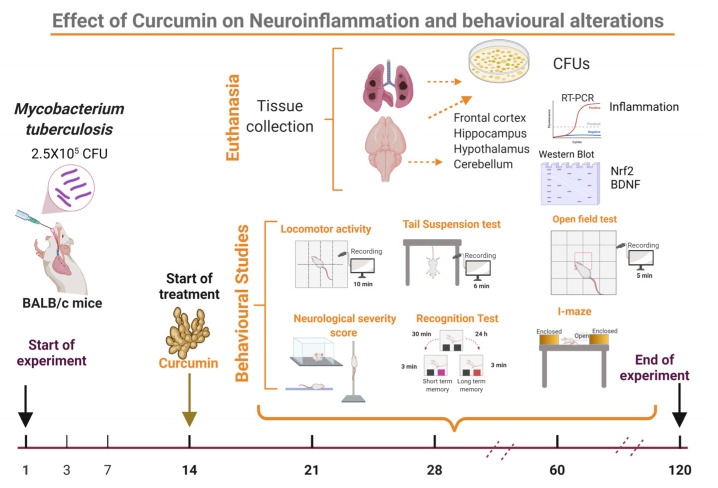
Experimental design to evaluate curcumin (CUR) treatment on neuroinflammation and behavioral alterations of TB mice. The therapy with CUR began on day 14. We administered CUR (16 μg/mL), and a group received saline solution (SS) as control. Different behavioral assessments were performed on days 21, 28, 60, and 120 after infection. Animals were euthanized after the behavioral tests, and the brain and lungs were taken to assess bacillary burdens. The cytokines gene expression was measured in the hypothalamus, hippocampus, cerebellum, and frontal cortex. Two separate experiments with *n* = 3 each were carried out for each of the measures. In addition, each experimental age group’s samples were examined individually. (Created with BioRender.com, accessed on 20 January 2021).

The effect of CUR treatment on the CNS inflammation of pulmonary TB mice was investigated. We assessed the treatment’s effect on pulmonary disease in the first stage of the study. For this part of the experiment, 84 mice were used (Supplementary Figure S1). Mice were treated after infection on day 14 and subsequently euthanized by exsanguination under anesthesia on days 21, 28, 60, and 120; lungs and brains were collected immediately to assess bacillary burdens by CFU counts, the left lung was used to determine the pneumonia area and survival was tracked throughout the trial (Figure 10). The second phase of the study looked at the effect of CUR on sickness behavior, behavioral abnormalities, and immunological response in various brain regions (Figure 11). We used 216 mice to meet this goal (Supplementary Figure S2). In agreement with The Mouse Brain in Stereotaxic Coordinates, the selected regions of the brain (hypothalamus, hippocampus, cerebellum, and frontal cortex) were promptly dissected by sectioning with a razor blade [99]. The hippocampus was found under the temporal cortex (Bregma: −1.06 mm, Interaural: 2.74 mm), while the cerebellum was discovered between the brainstem and the 4th ventricle’s lateral recess (Bregma: −5.84 mm, Interaural: −2.04 mm). The frontal cortex was acquired as the anterior section of the brain’s frontal lobes (Bregma: 1.70 mm, Interaural: 5.50 mm,) and the hypothalamus was localized as the area lateral and medial to the fornix (Bregma: −1.06 mm, Interaural: 1.62 mm). The material was immediately frozen by immersion in liquid nitrogen and used to measure cytokine gene expression using RT–PCR and protein expression by western blot. Various behavioral studies were carried out throughout the course of pulmonary TB. The tests performed included sickness behavior (bodyweight loss, LMA and food intake), anxiety-like behavior, unconditioned fear, NSS, depression-like behavior, and short and long-term memory. If respiratory insufficiency, exacerbated cachexia, or complete immobility was seen, the animals were humanely euthanized under pentobarbital anesthesia. Two separate experiments were performed.

### 4.5. Curcumin Preparation and Administration

CUR powder was dissolved in DMSO to make a solution with a final concentration of 0.05% of DMSO. After 14 days of infection, groups of three mice based on euthanasia time in two independent experiments were treated with 16 or 32 μg/mL of CUR administered by intraperitoneal route (100 μL) three days per week (Monday, Wednesday, and Friday). Control mice received 100 μL of saline solution with 0.5% of DMSO.

### 4.6. Colony-Forming Units (CFU) in Lungs and Brain of TB Mice

Bacterial colonies were counted in the right lungs and right hemisphere of the brains of six mice at each time point of two separate experiments. First, the lungs and brains were homogenized in sterile tubes containing 1 mL of isotonic saline solution using a FastPrep homogenizer (MP Biomedicals). Four homogenate dilutions were spread onto Bacto Middlebrook 7H10 agar supplemented with OADC on triplicate plates. CFU count was performed over 21 days of incubation at 37 °C and 5% CO_2_ [47,57].

### 4.7. Determination of Lung Affected Area by Pneumonia

For the histological/morphometric study, the left lung of six mice per group was perfused (IT) with 100% ethanol. Dehydrated parasagittal portions were embedded in paraffin (Oxford Labware, St. Louis, MO, USA) sectioned at a width of 3 µm and stained with H&E. An automated image analyzer system was used to make a reconstruction of the lungs and to measure the complete lung surface area affected by pneumonia. (Q Win Leica, Milton Keynes, UK). The measurements were performed blind, and the results were presented as mean values ± SEM from 3 individual mice in two separate experiments.

### 4.8. Expression of Cytokine Determined by RT-PCR

The RNeasy Mini Kit was used to isolate mRNA from the hippocampus, hypothalamus, cerebellum, and frontal cortex of six CUR-treated and control TB mice at each time point, following the manufacturer’s instructions. Spectrophotometry (260/280) and agarose gels were used to assess the quality and amount of RNA. One hundred ng of RNA, the oligo dT, and the Omniscript kit (Qiagen) were used to reverse-transcribe the mRNA. The 7500 RT-PCR equipment (Applied Biosystems, San Francisco, CA, USA) and the Quantitec SYBR Green Mastermix kit were used for real-time PCR (Qiagen). In each PCR cycle, negative controls were added. Using the Primer Express software (Applied Biosystems), specific primers for genes encoding glyceraldehyde-3-phosphate dehydrogenase (GAPDH) as a housekeeping gene and TNFα, IFNγ, IL12 were designed (Table 1). Initial denaturation at 95 °C for 15 min was followed by 40 cycles at 95 °C for 20 s, 60 °C for 20 s, and 72 °C for 34 s. Each sample was examined twice. The 2^−(^^△△Ct)^ technique calculates the fold change in gene expression [100].

### 4.9. Study of Nrf2 and BDNF by Western Blot Assay

To study if Nrf2 and BDNF are involved in protecting the brain mediated by CUR of TB mice, the expressions of these proteins were determined by western blot as described elsewhere [101]. The hippocampus and frontal cortex were dissected quickly and homogenized in HB buffer (20 mM HEPES pH 7.4, 1 mM EDTA, 1 mM DTT, 1 mM PMSF, 1 μg/mL pepstatin A, 1 μg/mL aprotinin, 1 μg/mL leupeptin and 1X phosphatase inhibitor cocktail 3) plus 0.5% Nonidet P40 and incubated on ice for 15 min. The homogenates were centrifuged at 850× *g* for 10 min at 4 °C. The supernatants were collected, as they were part of the cytoplasmic fraction (F1), and the pellets were resuspended in HB buffer, incubated on ice for 10 min, followed by the addition of 15 μL of 10% Nonidet P40 and incubated for 5 min. The samples were centrifuged at 14,000× *g* for 2 min at 4 °C, the supernatants were collected and added to the cytoplasmic fraction (F2) and the pellets were washed three times with 300 μL of HB buffer resuspended in complete lysis buffer (20 mM HEPES, 1.5 mM MgCl_2_, 0.2 mM EDTA, 20% Glycerol, 420 mM NaCl and 1 mM DTT) plus 1 mM PMSF, 1 μg/mL pepstatin A, 1 μg/mL aprotinin, 1 μg/mL leupeptin and 1X phosphatase inhibitor cocktail 3, vortexed on ice and incubated for 30 min at 150 rpm. The samples were vortexed for 30 s and centrifuged at 14,000× *g* for 10 min at 4 °C, and the supernatant (nuclear-enriched fraction) was collected. Protein quantification was determined by the Lowry method in the cytoplasmic fractions and used in western blot analysis. Briefly, 80 µg of protein of cytoplasmic fraction were loaded and separated in 10% SDS polyacrylamide gel electrophoresis and transferred to polyvinylidene fluoride (PVDF) membranes (Millipore, Bedford, MA, USA). Membranes were blocked using 5% low-fat milk in TBS plus 0.1% Tween 20 (TBS-T) for 2 h at room temperature with slight agitation. Blots were then incubated with anti-Nrf2 (1:500), anti-BDNF (1:500) or anti-β-tubulin (1:15,000) at room temperature overnight. Membranes were washed three times (10 min) with TBS-T. A horseradish peroxidase-conjugated secondary polyclonal antibody anti-rabbit (1:10,000), anti-goat (1:10,000) and anti-mouse (1: 10,000) was then added for 2 h at room temperature and after extensive washing with TBS-T. Bands were detected using the Immobilon Western kit (Millipore Co, Billerica, MA, USA), and the images were obtained with the imaging system Fusion Solo S (Eberhardzell, Biberach, Germany). To detect two or more proteins, the membranes were washed with a stripping solution (containing 0.2 M glycine, 0.1% SDS and 1% Tween 20, pH 2.2). Area values were obtained from each band’s pixel densities (PD) relationship. Area values of each group were standardized to the area value of the control group (value = 1). Data are expressed as relative optical density using ImageJ software [102].

### 4.10. Behavior Tests in Infected TB Mice

The approach for conducting behavior tests in the mouse model of progressive lung TB was previously reported [47,57]. Animals were exposed to the test environment 24 h before it was performed. To avoid any habituation, groups of mice were only examined once at the indicated time periods after treatment. All behavioral tests were conducted during the first 4 h of the light cycle’s dark phase. From these recordings, a blind observer examined and documented the results.

#### 4.10.1. Sickness Behavior Study

We evaluated LMA, food consumption, and weight loss to determine sickness behavior. In an open environment, the effect of *Mtb* lung infection on LMA was assessed by measuring the mice’s movement time for 10 min. The percentage of movement throughout the 10 min is shown in the corresponding graph. The amount of food fed to the mice was weighed twice a week to assess food intake, and the total amount of food consumed by the mice was determined. The data are provided in grams per mouse each day. The weight loss of *Mtb*-infected mice was calculated from day one to day 120 after infection. The animals were weighed every week, and their weight reduction was documented. The data are expressed in grams of body weight.

#### 4.10.2. Depression-like Behavior Study

The tail suspension test [103] was used to assess depression-like behavior. Animals were hung from the tail for 6 min in a tripod 30 cm high, and their activity was monitored, focusing on time spent in behavioral despair by mice. During those 6 min, the animal’s behavioral despair was recorded.

#### 4.10.3. Anxiety-like Behavior Study

The elevated I-maze, which is a modification of the elevated plus-maze model of anxiety in mice [59], was used to assess anxiety-like behavior. The I-maze is made up of three sections: a straight wooden path that resembles the English letter “I,” two enclosed spaces (close arms) at both ends of the “maze,” and an open area in the middle of the two enclosed regions. Percent TO, pHDIPS, uHDIPS, and SAP were measured after the animals were observed for 5 min.

#### 4.10.4. Unconditioned Fear Study

The open-field test, which compares the intrinsic dread of being in the central open area with the desire to explore different settings, was used to measure unconditioned fear and anxiety [104]. We used the same open field as in the LMA test, but we recorded a video from the top in a 5-min session and analyzed how much time was spent on the outside area. The data are provided as a percentage of the total time spent by the mice in the outside area.

#### 4.10.5. Neurological Outcome Study

Infected mice’s motor function and reflexes were assessed using a modified neurologic severity score (NSS) [105] and have been reported elsewhere [47,57]. These modifications included assessments to test the animals’ senses. Test characteristics are shown in Supplementary Table S1, except for hypomobility, motor impairment, and balance, which were evaluated as weak (1), moderate (2), or strong (3), they were valued as absent (0) or present (1). The highest possible score was 31 (indicating neurological damage). The usual rate was between 3 and 6 percent (standard).

#### 4.10.6. Memory Damage Study

After a lung infection with *Mtb*, the Object Recognition Test was used to measure memory and learning [106]. We used this test to assess both short- and long-term memory. We placed the animal in an open field for 10 min without any objects to familiarise it with the surroundings during the initial habituation phase. Two identical objects (objects A) were put in different places after 24 h, and the animal was left in the box for 3 min. Short-term memory was tested 30 min later. For this, we placed an object A (familiar item) in one location and a new object in the other (object B), and we tallied the interactions with both objects (the animal sniffs or touches the object with its front legs) for 3 min. After 24 h, the long-term memory was tested, item B was replaced with a new object (C), and the procedure was repeated. The discrimination ratio, the fraction of the novel object interaction of the total interactions, displayed the results obtained as shown in the following equation [106].

(1) $$Discrimination\;ratio=\frac{novel\;object\;interaction}{total\;interaction\;with\;both\;objects}$$

### 4.11. Statistical Analysis

The mean and standard error of the mean (SEM) from 3 individual mice in two separate experiments represents the data. All data collecting was performed in random order. The Shapiro–Wilk normality test was used to determine the normality of the data. The survival curve was analyzed using the logrank test. The statistical significance of the bacilli load, body weight, locomotor activity, and behavioral tests were determined using two-way ANOVA, followed by Dunnett’s multiple comparisons test or Tukey’s multiple comparisons test (comparison of each group to the saline control), as described in the related text. Unpaired *t*-tests analyzed Western blot assays. The cytokine expression was analyzed with the mixed-effects model. For all experiments, statistical significance was established at *p* < 0.05. GraphPad Prism was used to conduct the statistical analysis. (v 9.1.1.225) (GraphPad, San Diego, CA, USA).

## 5. Conclusions

**Figure 12:**
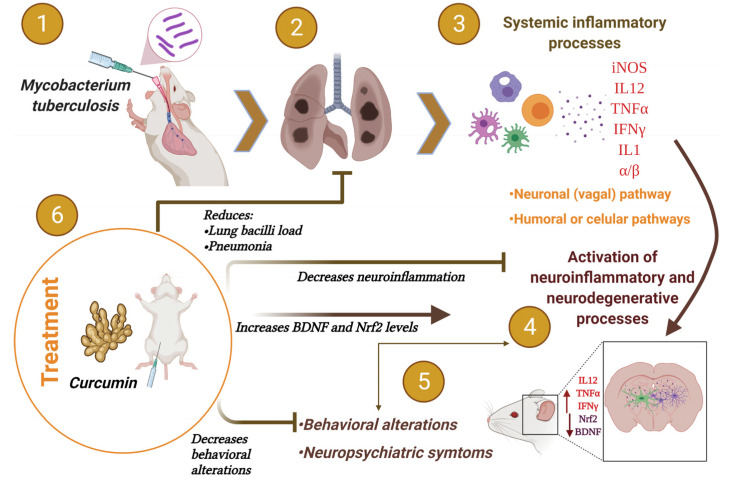
In a mouse model of pulmonary TB, curcumin (CUR) treatment slightly increased Nrf2 and BDNF levels, reduced neuroinflammation, behavioral and neuropsychiatric symptoms as well as lung disease. (1) Intratracheal lung infection with *Mtb* (2) produced the development of active disease in the mice. (3) Intense inflammation in the lungs caused by an immunological response to mycobacteria promotes neuroinflammation via humoral and neuronal pathways, (4) characterized by increased production of various cytokines (5), and decreased Nrf2 and BDNF levels, which lead to behavioral changes and neuropsychiatric symptoms such as depression and anxiety. (6) Due to the anti-inflammatory, antioxidant and antibacterial actions of CUR, the treatment decreased lung disease and cytokine generation in the brain, slightly increased Nrf2 levels in the frontal cortex and hippocampus and increased BDNF levels in the hippocampus. All these changes reduced the behavioral abnormalities in the TB animals. Therefore, CUR might be used as a coadjuvant treatment in TB chemotherapy. Created with BioRender.com (accessed on 20 January 2022).

CUR has a significant antibacterial and anti-inflammatory effect. In the present work, we showed that the administration of CUR in a murine model of pulmonary TB decreased the bacillary load of the lung, reduced the area of pneumonia, and tended to improve the survival of infected animals. Furthermore, CUR treatment decreased TNFα, IFNγ, and IL12 gene expression in the hippocampus, cerebellum, hypothalamus, and frontal cortex in the brain of mice infected intratracheally with *Mtb*. There was a tendency to increase Nrf2 levels in the frontal cortex and hippocampus. The results also showed a slight increase in BDNF levels in the frontal cortex. In the hippocampus, BDNF levels increase significantly. The treatment reduced the behavioral changes in tuberculous animals, such as sickness behavior, depression, anxiety, neurological damage, unconditional fear and memory damage. Thereby CUR could be used as an adjuvant in the TB treatment (Figure 12).

## Figures and Tables

**Table 1:** The primers’ sequences used to evaluate gene expression.

Gene	Forward	Reverse
GAPDH	5′-CATTGTGGAAGGGCTATGA-3′	5′-GGAAGGCCATGCCAGTGAGC-3′
TNFα	5′-GCCGAGAAAGGCTGCTTG-3′	5′-TGTGGCTTCGACCTCTACCTC-3′
IFNγ	5′-CCTCAACTTGGCAATCTCATGA-3′	5′-GGTGACATGAAAATCCTGCAG-3′
IL12	5′-GGATGGAAGAGTCCCCCAAA-3′	5′-GCTCTGCGGGCATTTAACAT-3′

## Data Availability

The data presented in this study are available on request from the corresponding author.

## Supplementary Materials

The following supporting information can be downloaded at: https://www.mdpi.com/article/10.3390/ijms23041964/s1.
