# Supplementary material for: Effect of Curcumin in Experimental Pulmonary Tuberculosis: Antimycobacterial Activity in the Lungs and Anti-Inflammatory Effect in the Brain
Source: Int J Mol Sci. 2022 Feb 10;23(4):1964. doi: 10.3390/ijms23041964 (PMC8876821; doi:10.3390/ijms23041964)
Supplement: Supplementary file 1 [file ijms-23-01964-s001.zip › ijms-1410756-supplementary.pdf]

Effect of Curcumin in Experimental Pulmonary Tuberculosis: Antimycobacterial Activity in the Lungs and Anti-Inflammatory Effect in the Brain

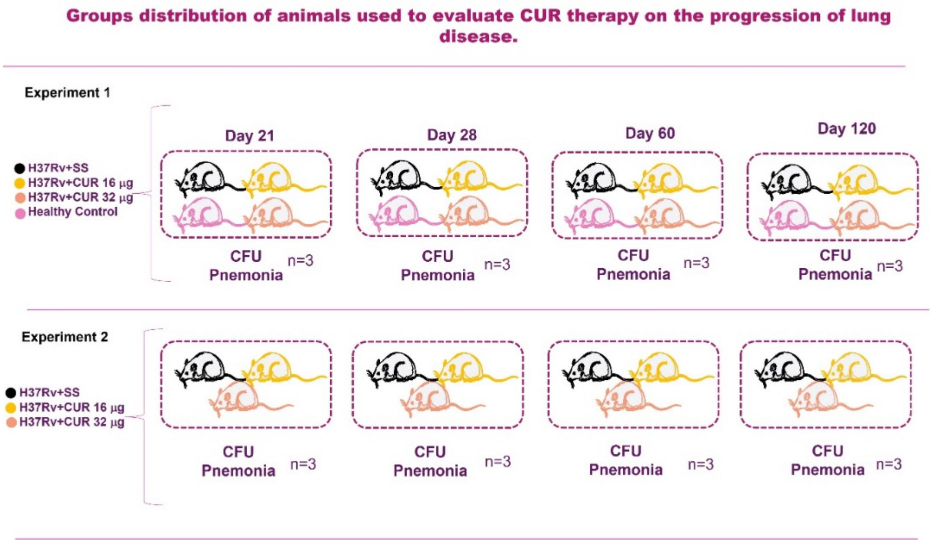

Figure S1. Animals' group's distributions used to evaluate CUR therapy on the progression of lung disease per experiment. Two independent experiments were done, and 84 mice were used (3/day/group/experiment).

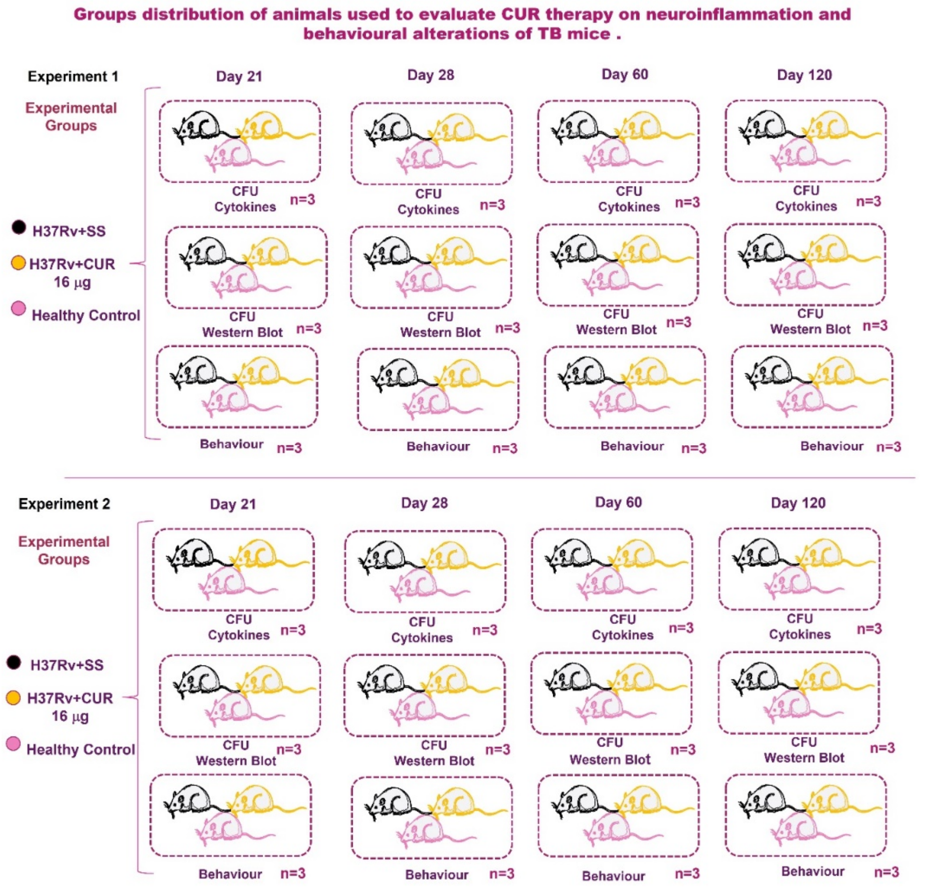

Figure S2. Animals groups distributions used to evaluate CUR treatment on neuroinflammation and behavioural alterations of TB mice per experiment. Two independent experiments were done, and 216 mice were used (9/day/group/experiment).

Table S1. Neurological Severity Score (NSS) for mice. Modified from Stahel et al., 2000 [1].

Task	Description	Points	
		**Success**	**Failure**
Hypomobility	Lower spontaneous mobility of the experimental mouse as compared with that of the external control when placed (30 s) on the top of the table. Scored 1,2 or 3 regarding locomotion, and speed and vigors of movements.	0	1,2,3
Lateralized posture	Abnormal body position characterized by a persistent tendency to recline sideward	0	1
Flattened posture	Abnormal body position characterized by slow movements and dragging the body along the tabletop	0	1
Hunched back	The persistent presence of a crouched posture	0	1
Piloerection	Persistent rise of the back hair.	0	1
Ataxic gait	The tendency to sway, rock, or lunch to the side as the animal proceeds forward.	0	1
Circling	Spontaneous or forced (gently pushing with one finger) walking consistently to one side.	0	1
Tremors	Presence of fine, repetitive, oscillatory movements observed during movement.	0	1
Twitches	Abrupt body jerks.	0	1
Convulsions	Repetitive twitches followed by extensions of the hind limbs.	0	1
Respiratory distress	Presence of increased, irregular, respiratory movements accompanied by breathing sounds.	0	1
Passivity	Characterized by the decreased behavioural response (struggle and escape) when the animals are covered with the hand to restrain movement.	0	1
Hyperreactivity	Characterized by the exaggerated behavioural response (struggle and escape) when the animals are covered with a hand to restrain movement.	0	1
Irritability	Characterized by aggressive posture and bitting behaviour exhibited by the animals when covered with a hand to restrain movement.	0	1
Ptosis	Closure of dropping of the upper eyes.	0	1
Urination	An excessive amount of urine on the animal's body and the tabletop	0	1
Decreased body tone	Characterized by relatively less resistance to compression or flaccidity of the abdominal muscles, determined by gentle compression of the sides of the animal between the lower thorax and pelvis using the thumb and index finger.	0	1
Forelimb flexion	Failure to extend one forepaw fully when the animal is held by the tail to a height of 10 cm and slowly lowered to observe symmetry in the outstretching of both forelimbs while the mouse reached the wire-mesh.	0	1
Decreased muscle strength	Characterized by decrease resistance when the animal is placed off the grid and gently drawn backwards by the tail.	0	1
Body rotation	Rolling along the axis of its body when the animal is held by the tail.	0	1
Motor incoordination	Characterized by decrease capacity to move and remain for at least 10 s on the inclined plane (45°) or to grasp the cord (30 cm above the tabletop) with limbs and tail, and to remain there for a least 10 s.	0	1,2,3
Absence of equilibrium	Characterized by a decreased capacity to remain for at least 10 s on a horizontal bar (1 cm wide). Four paws have to be on the bar.	0	1,2,3
Hypoalgesia	Lack of behavioural response when an arterial claw is placed at 1 cm from the base of the animal's tail.	0	1
Hyperalgesia	Exaggerated behavioural response (disproportional vocalization and bitting) when an arterial claw is placed at 1 cm from the base of the animal's tail.	0	1
Sensitivity in the whiskers	Behavioural response when animal's moustaches are touched with a hyssop	0	1
Sensitivity in the abdomen	Behavioural response when the animal in gently touched in the abdomen.	0	1
Olfactory sensitivity	Behavioural response when hyssop with alcohol is placed in the noise of the animal	0	1
		**Global Score**	**31**

Reference

1. Stahel, P.F.; Shohami, E.; Younis, F.M.; Kariya, K.; Otto, V.I.; Lenzlinger, P.M.; Grosjean, M.B.; Eugster, H.-P.; Trentz, O.; Kossmann, T.; et al. Experimental closed head injury: Analysis of neurological outcome, blood-brain barrier dysfunction, intracranial neutrophil infiltration, and neuronal cell death in mice deficient in genes for pro-inflammatory cytokines. *J. Cereb. Blood Flow Metab.* **2000**, *20*, 369–380, doi:10.1097/00004647-200002000-00019.
